# Design of the Prevention of Adult Caries Study (PACS): A randomized clinical trial assessing the effect of a chlorhexidine dental coating for the prevention of adult caries

**DOI:** 10.1186/1472-6831-10-23

**Published:** 2010-10-05

**Authors:** William M Vollmer, Athena S Papas, James D Bader, Gerardo Maupomé, Christina M Gullion, Jack F Hollis, John J Snyder, Jeffrey L Fellows, Reesa L Laws, B Alexander White

**Affiliations:** 1 Center for Health Research, 3800 N. Interstate Blvd, Portland, Oregon 97227, USA; 2 Tufts University School of Dental Medicine, 1 Kneeland St., Boston, MA 02111, USA; 3 Dept. of Operative Dentistry CB#7450, University of North Carolina, Chapel Hill NC 27599- 7450, USA; 4 Indiana University School of Dentistry, 415 Lansing St., Indianapolis IN 46202, USA; 5 Permanente Dental Associates, 500 NE Multnomah St, Suite 100, Portland OR 97232, USA; 6 DentaQuest Institute, 2400 Computer Dr, Westborough MA 01581, USA

## Abstract

**Background:**

Dental caries is one of the primary causes of tooth loss among adults. It is estimated to affect a majority of Americans aged 55 and older, with a disproportionately higher burden in disadvantaged populations. Although a number of treatments are currently in use for caries prevention in adults, evidence for their efficacy and effectiveness is limited.

**Methods/Design:**

The Prevention of Adult Caries Study (PACS) is a multicenter, placebo-controlled, double-blind, randomized clinical trial of the efficacy of a chlorhexidine (10% w/v) dental coating in preventing adult caries. Participants (n = 983) were recruited from four different dental delivery systems serving four diverse communities, including one American Indian population, and were randomized to receive either chlorhexidine or a placebo treatment. The primary outcome is the net caries increment (including non-cavitated lesions) from baseline to 13 months of follow-up. A cost-effectiveness analysis also will be considered.

**Discussion:**

This new dental treatment, if efficacious and approved for use by the Food and Drug Administration (FDA), would become a new in-office, anti-microbial agent for the prevention of adult caries in the United States.

**Trial Registration Number:**

NCT00357877

## Background

Although children are the primary recipients of caries-prevention programs in the United States (US), a recent meta-analysis concluded that older adults experience caries at the same or even higher rates [1]. Indeed, dental caries is one of the primary causes of tooth loss among adults [2-4], and is estimated to affect a majority of Americans aged 55 and older [5]. Root caries in particular is one of the most common chronic infectious diseases of midlife. Among Americans aged 45-64, root caries is more prevalent (at 35%) [6] than chronic joint symptoms (32%), hypertension (32%), arthritis (29%), symptoms of mental illness (14%), heart disease (13%), and diabetes (10%) [7]. Data from a largely employed population with dental insurance found that mean annual spending on adult dental care peaks between ages 55 and 64, at about $700 per capita, and that caries-related treatment accounts for about half of these expenditures [8].

Adult caries can be particularly devastating in disadvantaged populations, such as the uninsured and certain racial and ethnic minority groups [5]. One subgroup that is particularly affected by dental caries is American Indians. Based on data from the Indian Health Service's 1999 oral health survey [9], 68% of 19-year-old American Indians have experienced caries, compared to 24% of non-American-Indian 15-19-year-olds. Currently only about half of Americans (and just 1 in 5 over age 65) have dental insurance [10,11]. While the literature on the impact of dental insurance on dental health is limited, it is clear that the uninsured have a higher level of edentulism (27% vs. 18.3% among insured) [12], a higher number of untreated caries [13], and a lower likelihood of visiting the dentist than the insured [14,15]. When they do see the dentist, the uninsured are more likely to receive a filling or extraction, or other acute care, and are somewhat less likely to receive preventive services.

Although several practices are recommended to prevent adult caries, a 2001 National Institutes of Health (NIH) consensus panel concluded that the evidence base for adult caries prevention is limited [16]. Everyone should be advised to floss daily and use fluoridated toothpaste 2-3 times per day. Adjunctive treatments may be used, including in-office fluoride gels, professionally applied fluoride varnish, or home use of prescription (5000 ppm) topical fluoride [17-19].

Chlorhexidine is an antimicrobial that may be an effective adjunctive treatment for caries prevention. To date, chlorhexidine rinse (0.12% w/v) has been approved for use to reduce gingivitis, but not for caries prevention. Chlorhexidine rinse, when used in an alternating sequence of daily use for one month, then weekly for 5 months, failed to reduce caries more than a placebo (quinidine) rinse over 5 years in a randomized study of 1101 adults 65 years and older [20]. In addition, continual use of chlorhexidine rinse may stain teeth.

Two placebo-controlled trials of the efficacy of a chlorhexidine (10% w/v) dental coating applied by a dental hygienist or dentist to prevent caries were reported in 2000 [21,22]. Possible therapeutic advantages of this formulation include a much higher chlorhexidine concentration and longer contact time with the tooth surface, which should intensify the anti-cariogenic effects of chlorhexidine. In addition, in-office application avoids patient compliance issues.

The first study enrolled 240 adults with xerostomia aged 40-80 with an elevated risk of dental caries[21]. Participants were randomly assigned to active treatment or to one of two control conditions (either a sham treatment that contained quinine hydrochloride to mimic the bitter taste of chlorhexidine or a placebo control) and followed for 13 months, with four initial weekly treatments followed by a fifth treatment at six months. Relative to placebo, the active treatment group had a 24.5% greater reduction in combined root and coronal caries increment (p = 0.03), a 40.8% reduction in root caries increment (p = 0.02), and a 14.4% reduction in coronal caries increment (p = 0.06). Although caries increment scores also were reduced for the active treatment relative to the sham groups (13.8%, 32.5%, and 2.2%), these reductions were not statistically significant (potentially because of the antimicrobial properties of the sham agent, quinine hydrochloride).

The second study enrolled 1,240 economically disadvantaged adolescents aged 11-13 years with a history of decay and an elevated level of *Streptococcus mutans *(250,000 cfu/mL). Participants were randomly assigned to one of four arms: active drug, a placebo coating plus best practice preventive care, a best practice preventive care control condition, and a usual care control condition. All participants were followed for three years. Participants in active and placebo-coating arms received four initial weekly treatments, followed by additional treatments at 12 and 24 months post- randomization. Participants also received up to two additional treatments each year, depending upon their level of salivary *S. mutans*. An intent-to-treat analysis found no treatment effect overall [22], but adherence to the study treatment was poor, possibly affecting the results. Unpublished secondary analysis in the per-protocol sample found significant reduction in caries among girls in the active treatment group compared to girls in control arms, as well as a gender difference in use of sugary carbonated drinks, which may have reduced efficacy.

The current manuscript describes the protocol for the Prevention of Adult Caries Study (PACS), which represents a third evaluation of the chlorhexidine dental coating and is being conducted as a "pivotal" study under US Food and Drug Administration IND #45,466. PACS is a randomized clinical trial being conducted over a 13-month observation period among adult participants from four diverse communities in the United States, each of which is served by distinctly different dental-care delivery systems. Of note, PACS includes a large uninsured population lacking regular access to dental care, the western Navajo Nation and Hopi populations in northern Arizona, and a large metropolitan community without a fluoridated water supply. The primary objectives of PACS are (1) to test the hypothesis that the chlorhexidine dental coating, compared to a placebo coating, will reduce dental caries increment in at-risk adults from baseline to the 13-month follow-up visit; and (2) to evaluate the cost and cost-effectiveness of using the treatment from patient, program, and provider perspectives.

## Methods/Design

PACS is a multicenter, placebo-controlled, double-blind, randomized clinical trial. Participating institutions include the Kaiser Permanente Center for Health Research in Portland, OR, which serves as the data coordinating center, and four clinical centers: Tufts University School of Dental Medicine (TUSDM) in Boston, MA; the Tuba City Regional Health Care Corporation (TCRHCC) on the Navajo Reservation in northern Arizona; the Dental Service of Massachusetts clinic in Southborough, MA (a part of Delta Dental of Massachusetts); and the Kaiser Permanente Dental Care Program and Permanente Dental Associates in Portland, OR. The study chair is located at Tufts University, and this center also is the location of the microbiology laboratory. The study is sponsored by the National Institute of Dental and Craniofacial Research (NIDCR). The product manufacturer, CHX Technologies Inc., provides the study treatments, a Good Clinical Practice (GCP) auditor (Schiff & Associates), and some additional funding and quality assurance of the product. Independent oversight of trial activities is provided by a Data and Safety Monitoring Board appointed by the NIDCR. The study was approved by the Institutional Review Boards of each participating institution and by the FDA, and all participants provided written informed consent.

### Study Population

The study population consists of individuals aged 18-80 years, having 20 or more intact teeth and deemed at increased risk of dental caries due to the presence of one or more cavitated lesions at screening (Table 1). We excluded participants for whom the study treatment would be contraindicated, as well as participants with health conditions that might affect the measurement of study outcomes or their ability to successfully complete the study. We targeted four communities with varying fluoride exposure, dental reimbursement, and overall risk of caries (Table 2). Financial incentives varied by site and included cash incentives, reimbursement of travel expenses and, for some individuals, reimbursement for the cost of required restorative care. Recruitment began in February, 2007 and concluded in August, 2008.

**Table 1:** Eligibility Criteria for PACS

Inclusion Criteria
* 18 years of age and older

* at least 20 intact natural teeth

* 2 or more lesions, one of which being a cavitated D2 or D3

* willing and able to provide informed consent

Exclusion Criteria

* pregnant or planning to become pregnant during the study (breastfeeding is permitted)

* use of fixed orthodontic appliances

* allergic to any of the ingredients of the study medication (chlorhexidine diacetate, Sumatra benzoin, alcohol, ammonio methacrylate copolymer type B, or triethyl citrate)

* long-term antibiotic therapy (defined as taking an antibiotic -- including low dose doxycycline (Periostat) - for 30 days or more in the past 3 months)

* currently taking any anti-fungal medication prescribed by a doctor or dentist

* a history of, or currently active, radiation therapy for cancers of the head or neck

* Sjögren's syndrome

* advanced periodontitis and in the clinical judgment of the examiner, there is the likelihood the participant will not have 20 natural teeth at the end of the study

* having ten or more teeth requiring restorative care at the time of the screening visit

* requires antibiotic prophylaxis for dental care

* remineralization therapy within one month of randomization (includes use of a fluoride varnish, 5000 ppm prescription fluoride toothpaste, a 0.12% chlorhexidine mouth rinse or gel, sodium fluoride mouth rinse, use of Xylitol products, by mouth, two or more times per day for four or more days per week (excluding Trident)

* investigator discretion

**Table 2:** Characteristics of PACS Clinical Center Populations

Study Center	Recr quota	Fluoride in water	Dental reimbursement	Est. caries prevalence versus US average
KP Portland	400	Partly	Pre-paid managed care	Same

Tufts	200	Yes	Majority uninsured	Same

Dental Service of MA, Southborough	200	Partly	Primarily prepaid fee-for-service	Same

Tuba City	200	Partly	Access to free care	Higher

All participants attended an initial screening visit to assess eligibility. Eligible participants then received necessary restorative care for all cavitated lesions prior to returning for a randomization visit, at which time additional baseline measurements (including a baseline caries examination) were obtained. Individuals who still had cavitated lesions at this point were referred back to their treating dentist for further restorative care prior to randomization, and the randomization visit was then repeated.

Eligible participants were randomized to receive five applications of either a chlorhexidine (10% w/v) or placebo dental coating over a seven-month interval. A computerized randomization process confirmed participant eligibility prior to issuing randomization assignments. The latter were stratified by clinical center and age and, within each strata, clustered in blocks of varying sizes. Participants and staff were blinded to treatment assignment. Overall, we randomized 983 participants, close to our goal of 1000.

### Schedule of Activities

Treatment and data collection occurred at seven visits during the study period (Table 3). We collected baseline data and determined study eligibility at both the screening visit (SV) and randomization (V1) visits, which had to occur within 4 months of each other. The first treatment application occurred immediately following randomization at the V1 visit. Three additional treatment applications took place on a roughly weekly basis at the second through fourth visits (V2- V4), followed by a fifth and final treatment application seven months post-randomization as part of the fifth visit (V5). The V5 visit also included a comprehensive outcome assessment, together with a dental caries examination. Active caries discovered at the V5 visit had to be restored before the final treatment application. The final data-collection visit (V6) was scheduled six months later, or approximately 13 months post-randomization. This visit included a comprehensive outcome assessment.

**Table 3:** Schedule of Study Visits

	SV (t_s_)	V1 (t_0_)	V2 (t_1_)	V3 (t_2_)	V4 (t_3_)	V5 (t_4_)	V5a (t_5_) Tufts only	V6 (t_6_)	V6a (t_7_) Tufts only
Target date		t_s + _4 mos	t_0_+7d	t_1_+7 d	t_2_+7 d	t_0_+7 mos	t_0_+10 mos	t_0_+13 mos	t_0_+19 mos

Allowable window			5-14 d	5-14 d	5-14 d	± 1 mo	± 1 mo	± 1 mo	± 1 mo

Eligibility questions	X	X^1^							

Exam for eligibility	X								

Demographic questions	X								

Informed consent	X	X^2^							

Dental restorations	X^3^					X^3^		X^3^	

Pregnancy test	X	X				X			

Acidic drink consumption questions	X				X	X		X	

Medical HX questions		X				X		X	

Oral Health HX questions		X						X	

Randomization		X							

Tooth Surface Exam		X				X		X	

Coating Application		X	X	X	X	X			

Adverse Events questions		X	X	X	X	X	X	X	X

Medication questions	X	X	X	X	X	X	X	X	X

Soft Tissue Exam		X				X		X	

Travel time/costs		X				X		X	

Care Utilization								X	

Candidiasis Lab Tracking	X	X	X	X	X	X	X	X	X

Medication Price Survey		X							

Microbiology Tufts only		X^4^				X^4^	X^4^	X^5^	X^5^

1 some eligibility information will be reassessed at V1

2 although not required, some study centers will use separate screening and randomization consent forms

3 following the screening visit (SV), any needed dental restorations are made prior to randomization (V1); additional restorative work, if needed, is performed following the dental examinations at the V5 and V6 visits

4  testing for presence and level of *S. mutans *and *C. albicans *in a subset of patients at the Tufts clinical center

5 additional testing for *S. mutans *and *C. albicans *may occur at V6 and V6a depending on results of earlier tests

Participants at the Tufts clinical center also were asked to take part in an optional substudy to assess the effect of the study medication on resistant (*S. mutans*) and opportunistic (*Candida albicans*) infections in the oral cavity. At each of the V1, V5, V5a, V6, and V6a visits, selected participants provided a sample of stimulated whole saliva for analyses of *S. mutans *sensitivity to chlorhexidine and swabs of the right and left buccal cheek mucosa for analyses of levels of *C. albicans*. Specimens were processed and analyzed by staff trained in Good Laboratory Practice (GLP) at the microbiology laboratory, which forwarded the results directly to the data coordinating center. A total of 143 Tufts subjects participated in this substudy.

### Study Treatments

Study treatments were applied in-office by a dental hygienist after a brief rubber cup prophylaxis with non-fluoridated paste and scaling as needed. The coatings were applied in two stages. In stage 1, the first coating was applied to all tooth surfaces in a given quadrant of the dentition. This was immediately followed by application of the second coating (labeled Stage 2 on the vials) to the same quadrant. The other three quadrants were similarly treated and the coatings were air dried to ensure proper hardening. All dental hygienists were trained and certified in the proper application of the coatings, and the entire process typically took 20 minutes to complete.

The stage 1 coating contains either chlorhexidine diacetate suspended in a solution of Sumatra benzoin and alcohol (the active treatment) or simply the Sumatra benzoin and alcohol solution (the placebo treatment). The second coating is a proprietary aqueous dispersion of inert methacrylate approved for use by the FDA under license K013671. This second coating lasts until it is abraded by hard foods or is brushed from the teeth, and is designed to give the chlorhexidine extended contact time on the enamel.

The component medications used in both the Stage 1 and Stage 2 coatings were made under Good Manufacturing Practices[23] in an FDA-approved facility. All treatments were packaged in a cardboard box containing ten 2-mL capped amber glass vials (sufficient for all 5 study treatments). Each vial contained either 1 mL of the Stage 1 coating or 1 mL of the Stage 2 coating. The box was labeled for PACS and had a tamper-proof seal. Stage 1 and Stage 2 vials had different colored labels, and each vial was labeled for one of the five visits. The treatment boxes were shipped overnight in refrigerated packaging from the study pharmacist to the clinical centers; thermometers measured trans-shipment temperatures. Upon receipt by the clinical centers, the treatment boxes were immediately refrigerated. Studies have demonstrated that the study treatments are stable at room temperature for longer than three months and at refrigerated temperatures for 30 months [24,25].

For participants in the active treatment arm, the mean dose of chlorhexidine at each application visit was expected to be approximately 33 mg (or 330 μL), so that the cumulative mean dose for participants completing all five applications was expected to be 165 mg.

### Blinding of Study Treatments

Active and placebo-coating materials were packaged identically and were distinguishable only by a numerical label. Each box of treatment material contained a complete supply of coatings for one person for the entire study. Individual vials, together with the box in which they were packaged, were affixed with the same label. The sequence of numerical codes was random with respect to likelihood that the package contained active or placebo coating. Packing was done by a central facility under contract to CHX Technologies Inc., and active and control boxes were prepared in separate production runs using label identifications (IDs) supplied by the data coordinating center. Independent verification of a random sample of boxes was done to confirm that the correct labels were assigned to the active and placebo boxes.

Since prior studies have shown that differences in taste or appearance between the study medication and placebo were imperceptible when substances were applied according to protocol, the likelihood of inadvertent unblinding of the patient, dental team, or other clinic staff is minimal. In the unlikely event that staff do become unblinded to the treatment status of a given participant, the nature of the treatment allocation process (as described above) means that the unblinding would be a single event and that nothing could be inferred about other participants' treatment assignments.

### Caries Examination and PACS Taxonomy

Caries were diagnosed visually by calibrated examiners using a Community Periodontal Index of Treatment Needs (CPITN) probe, an unblemished, non-magnifying plane mirror; and standard dental operating light and chair. Use of loupes was according to local practice. The participants' teeth were dried for five seconds with an air/water syringe to better enable the examination of tooth surfaces. At the request of the FDA, radiographs were not used in diagnosis. Each central, lateral, and cuspid tooth was deemed to have five coronal (including the incisal) surfaces and four root surfaces. Examiners made only one diagnostic judgment per tooth surface.

Two initial 4-day training and calibration sessions were held at the two East Coast and West Coast sites with a gold standard examiner. These were followed by three recalibrations at roughly nine-month intervals.

The PACS taxonomy of adult dental caries used the nomenclature of Pitts and Fyffe [26] that identifies three basic stages of lesions: non-cavitated lesions (D1); lesions where the cavitation extends into, but not through, the enamel (D2); and cavitated lesions that involve the dentine (D3). (A modified taxonomy that does not include the D3 score is used for root surfaces.) Each stage of decay involves different and distinct treatment implications in terms of cost, staff time, and management. The D1, D2, and D3 designations are well understood by the community dentists in all four PACS clinical centers. This taxonomy has been used by Banting et al [21]. Papas et al.[27] and Chesters et al. [28] to detect caries incidence. The descriptors for the D1, D2, and D3 classifications in PACS are adapted from the International Caries Detection and Assessment System (ICDAS) II [29], as shown in Table 4. ICDAS II established objective clinical signs that have been associated with severity levels of dental caries (particularly the non-cavitated lesion) and verified histologically, and uses a 2-digit numeric code to classify each tooth site. The PACS taxonomy does not make a number of distinctions that are in the ICDAS II taxonomy. Instead, we have collapsed codes as follows:

**Table 4:** The PACS Taxonomy for Scoring Tooth Surfaces

PACS code	Definition	Corresponding ICDAS codes
S	sound or pits-and-fissures sealant on sound surface	00, 10, 20

D1	non-cavitated lesion on otherwise sound or sealed surface	01, 02, 11, 12, 21, 22

D2	cavitated lesion on otherwise sound or sealed surface	03, 04, 05, 13, 14, 15, 16, 23, 24, 25

D3	cavitated lesion extending into the dentine on an otherwise sound or sealed surface	06, 26

F	filling on otherwise sound or sealed surface	30, 40, 70, 80

FD1	non-cavitated lesion on surface already having a filling	31, 32, 41, 42, 71, 72, 81, 82

FD2	cavitated lesion on surface already having a filling	33, 34, 35, 43, 44, 45, 46, 73, 74, 75, 83, 84, 85

FD3	cavitated lesion extending into the dentine on a surface already having a filling	36, 76, 86

C	full crown on surface otherwise sound or sealed	50, 60

CD1	non-cavitated lesion on surface already partially covered by a crown	51, 52, 61, 62

CD2	cavitated lesion on surface already partially covered by a crown	53, 54, 55, 63, 64, 65

CD3	cavitated lesion extending into the dentine on a surface already partially covered by a crown	56, 66

Y	unscorable or invisible surface (e.g., unexposed root)	96, 99

M	surface on missing tooth	97, 98

• The first digit codes 0, 1, 2 are collapsed into S (sound or sealed surface), 3, 4, 7, 8 are collapsed into F (filled), and 5, 6 are collapsed into C (crowned)

• The second digit codes 1 and 2 were collapsed into a single D1 code (uncavitated lesion). This was done in recognition of the fact that it is difficult to train dentists in detecting pre-clinical caries lesions.

• The second digit codes 3 and 4 were collapsed into a single D2 code (cavitated lesion). This was done because any degree of cavitation is generally regarded as requiring intervention.

• The second digit codes 5 and 6 were collapsed into a single D3 code (cavitated lesion into dentin).

For purposes of our primary analysis, calls that require intervention are treated identically, so the D2 and D3 calls are collapsed into one category.

### Primary Study Outcome

The primary study outcome is the **net caries increment **(root and coronal surfaces combined) from baseline (V1) to end-of-study (V6), scored as the number of changes recorded from V1 to V6, including reversals. This measure permits only one transition per tooth surface (the V5 visit is used only to achieve a more efficient multiple imputation, see below) and allows for theoretically plausible reversals (e.g., from D1 to sound) as well as for implausible reversals that presumably result from measurement error in one of the calls (e.g., D2 to sound). Three additional measures of caries increment (Table 5) will be included as secondary outcomes, to give a comprehensive view of demineralization and remineralization.

**Table 5:** Analytical Models of Caries Increment

Model	Description
Crude increment	One event (change) per tooth surface is counted; no reversals are counted

Cumulative crude increment	Multiple events are possible on a tooth surface; no reversals are counted

Net increment	One event per tooth surface is counted; reversals are included

Cumulative net increment	Multiple events per tooth surface are possible; reversals are included

The net caries increment is computed as the sum, across tooth surfaces, of transition scores (weights) associated with 121 pre-defined transitions in tooth-surface integrity (Table 6). Our preliminary weighting scheme, as shown in the table, assigned a range of -2 to +2, with transitions to a worse status receiving positive weights and transitions (reversals) to a better status receiving negative weights. Transitions that reflect no change (e.g., D1 to D1), a change to treated status (e.g., D2 to F or C), or to or from an unscorable status (Y or M) are scored 0 and hence effectively excluded from analysis. Remineralization from D1 to S is considered a plausible reversal (-1), but not from D2 or D3. These tooth-surface transitions are similar to those used by Chesters et al. [28] in their fluoride-intervention study in young adolescents. Like Chesters, we recognized that reversals from cavitated lesions (bolded in Table 6) are not plausible. After completion of bootstrapping analyses of a caries increment dataset from another study (Gullion, personal communication, 2009), we concluded that the optimal way to handle unlikely or implausible transitions is to ignore them (i.e., assign a weight of zero). Hence for our primary analysis the bolded values in Table 6 will be ignored.

**Table 6:** Transition Weighting Matrix

	13-month Visit (V6)											
**Baseline** ** Visit (V1)**		**S**	**D1**	**D2/D3**	**F**	**FD1**	**FD2/FD3**	**C**	**CD1**	**CD2/CD3**	**Y**	**M**

	**S**	0	1	2	2	2	2	0	0	0	0	0
	
	**D1**	-1	0	1	1	1	1	1	1	1	0	0
	
	**D2/D3**	**-2**	**-1**	0	0	1	0	0	0	0	0	0
	
	**F**	**-2**	**-1**	**0**	0	1	2	0	1	2	0	0
	
	**FD1**	**-2**	**-2**	**-1**	-1	0	1	1	1	1	0	0
	
	**FD2/FD3**	**-2**	**-2**	**-2**	0	**-1**	0	0	0	0	0	0
	
	**C**	**-2**	**-1**	**0**	0	1	2	0	1	2	0	0
	
	**CD1**	**-2**	**-2**	**-1**	-1	0	1	-1	0	1	0	0
	
	**CD2/CD3**	**-2**	**-2**	**-2**	**-2**	**-1**	0	0	**-1**	0	0	0
	
	**Y**	0	0	0	0	0	0	0	0	0	0	0
	
	**M**	0	0	0	0	0	0	0	0	0	0	0

KEY

S = sound or pits-and-fissures sealant on sound surface (ICDAS codes 00,10,20)

D1 = non-cavitated lesion on otherwise sound or sealed surface (ICDAS codes 01,02,11,12,21,22)

D2/D3 = cavitated lesion on otherwise sound or sealed surface (ICDAS codes 03,04,05,06,13,14,15,16,23,24,25,26)

F = filling on otherwise sound or sealed surface (ICDAS codes 30,40,70,80)

FD1 = non-cavitated lesion on surface already having a filling (ICDAS codes 31,32,41,42,71,72,81,82)

FD2/FD3 = cavitated lesion on surface already having a filling (ICDAS codes 33, 34, 35, 36, 43, 44, 45, 46, 73, 74, 75, 76, 83, 84, 85, 86)

C = full crown on surface otherwise sound or sealed (ICDAS codes 50,60)

CD1 = non-cavitated lesion on surface already partially covered by a crown (ICDAS codes 51,52,61,62)

CD2/CD3 = cavitated lesion on surface already partially covered by a crown (ICDAS codes 53,54,55,56,63,64,65,66)

Y = unscorable or invisible surface (e.g., unexposed root) (ICDAS code 96,99)

M = surface on missing tooth (ICDAS code 97,98)

NOTE: some of the ICDAS codes listed may be specific to children, and are included only for completeness.

### Other Study Measures

PACS assessed a number of safety measures and predictors of caries increment at baseline.

#### Medical History

At V1, V5, and V6, participants completed a medical history questionnaire indicating whether they had ever been diagnosed or treated for a series of common medical conditions. An additional medical-eligibility questionnaire was asked at the screening visit.

#### Oral Health History

At V1 and V6, participants completed a brief questionnaire regarding their oral hygiene practices and dental history.

#### Soft Tissue Examination

Participants were examined for abnormalities of the oral mucosa at V1, V5, and V6. An assessment for candidiasis was part of this examination. This assessment also provides additional data to the microbiology substudy at the Tufts clinical center.

#### Adverse Events

Starting at V1, participants were asked if they had experienced any adverse events since the last visit. Additionally, at the treatment visits they were asked if they had experienced any adverse events during the treatment application. All serious adverse events (SAEs) were immediately reported to the data-coordinating center, the Project Office, the Study Chair and CHX Technologies Inc.; CHX Technologies Inc., in turn, notified the FDA. The respective Institutional Review Boards of each institution were also notified of adverse events.

#### Medication Use

Prescription medication use was tracked at each clinic visit.

#### Demographic Information

Demographic information was collected as part of the screening visit.

#### Pregnancy Status

Pregnant women and women who were planning to conceive during the course of the study were excluded from participation. As a safety precaution, we confirmed pregnancy status again at V5 and pregnant women did not receive the final treatment application.

#### Acidic Drink Consumption

The consumption of acidic beverages can prematurely erode the protective coating that was applied in stage 2 of the treatment application process. Participants were asked to avoid drinking such beverages for three days following treatment application, and we asked about typical consumption of acidic beverages at the SV, V4, V5, and V6 visits.

#### Intervention and Dental Care Costs

Program cost and cost-effectiveness analyses of the chlorhexidine coating will be assessed from patient and dental plan perspectives [30]. Study staff conducted comprehensive chart reviews to document all dental-care procedures (CDT and local codes), procedure dates, billed claims, amount paid by insurance, and insurance plan type for encounters of study participants from the randomization date to V6. Procedures and clinical service costs related to SAEs were noted. We obtained clinical staff earnings and clinic facility space to estimate provider training and service delivery costs. We also included participant travel time and costs for each clinical visit. These data will be used to calculate the incremental net cost-effectiveness per prevented caries increment of the intervention, compared to placebo, for total and restoration-related dental care expenditures. All costs will be expressed in a reference year values (e.g., 2009 dollars). Expenditures and outcomes occurring in month 13 will be discounted to present value terms using a financial discount rate for the medical-care service sector. Sensitivity analyses will be conducted by varying key model inputs, and sub-group analyses will be conducted if supported by the data. We also conducted a price opinion survey at SV to estimate patients' willingness to pay for the service and to support a cost-benefit analysis of the intervention [31]. TCRHCC participants receive free out-of-pocket care and were excluded from the price survey.

### Quality Control

In addition to being trained as caries examiners and recorders, staff were centrally trained and certified in all aspects of study operations, including questionnaire administration, data entry, and application of the dental coatings. Initial training occurred prior to the start of randomization, with recertification occurring roughly annually. The data-coordinating center monitored study progress on an ongoing basis and generated regular trial monitoring reports for review by the Steering Committee and the Data Safety Monitoring Board (DSMB).

The data-coordinating center used a secure, web-based application for data entry and management. This application incorporated real-time error checking and quality assurance at the time of data entry and prompted clinical center staff about potentially erroneous data during data entry. Additional "back-end" checks were performed at the data-coordinating center.

In addition to the routine trial monitoring reports generated by the data-coordinating center, Internet-enabled reporting tools allowed authorized clinical center staff to access additional reports and edit data on an ad hoc basis. The data-coordinating center maintained an electronic audit trail of all errors and error resolutions.

PACS employed three types of site visit monitoring. CHX Technologies Inc. conducted their own ongoing monitoring. Schiff & Associates, an outside contractor employed by CHX Technologies Inc., conducted three independent monitoring visits at each clinical center--at the beginning, middle, and end of the study. And finally, the data-coordinating center conducted annual site visits at each clinical center to review all aspects of quality assurance, as defined in GCP [32].

### Data Analysis

Analyses will be carried out in three samples. The **intent-to-treat (ITT) **sample includes all randomized participants, regardless of their adherence to the protocol, and classifies them according to their assigned treatment group. The **per-protocol **sample consists of participants who received all five applications of their assigned study treatment, or who were removed from treatment due to an adverse event, and did not deviate from the protocol in any significant way that could have affected the results. The **safety **sample consists of all participants who received at least one study treatment. These individuals are classified according to the actual treatment received.

In order to include all randomized participants in the ITT sample, we will use multiple imputation [33] to impute missing caries examination data for the V6 (final) visit. The planned primary- outcome analysis will be repeated in exactly the same way in each of the imputed datasets, and the results combined using Rubin's [34] rules to produce the adjusted estimates and statistics from which inferences will be drawn.

All statistical hypothesis tests will be performed with two-sided type I error level of α=0.05. Interaction terms, when included in the model (e.g., treatment-by-site), will be assessed at α=0.10. No adjustment for multiple testing is planned.

#### Primary Analysis

The primary efficacy analysis will be in the ITT population, using analysis of covariance (ANCOVA), with treatment group, clinical center, and examiner included as categorical factors, and age and age-squared as continuous covariates. We will use rank-normalized net caries increment scores in this analysis. We next will evaluate whether an interaction exists between clinical center and treatment and, if so, will estimate and test center-specific treatment effects.

#### Secondary Analyses

##### Secondary Aim 1

We will test whether the effect of the study medication differs within subgroups of the ITT sample defined by: baseline decayed filled surfaces (DFS) (median split), age (quartile groups), sex, and whether these added covariates account for any clinical center differences observed in the primary analysis.

##### Secondary Aim 2

We will evaluate incidence and increment of root and coronal caries in ITT participants by age, socioeconomic status (education, income), dental care setting (managed care, fee-for-service, etc.), and health-related profile.

##### Secondary Aim 3

We will evaluate the impact that various models of caries increment and detection criteria have on incidence and prevalence estimates of this most common adult disease.

##### Secondary Aim 4

We will evaluate the effect of the chlorhexidine (10% w/v) dental coating on resistant (*S. mutans*) and opportunistic (*C. albicans*) infections in the oral cavity using the subset of Tufts participants who are enrolled in the substudy.

#### Sensitivity Analyses

We will conduct a number of sensitivity analyses to evaluate the robustness of the primary efficacy analysis with respect to dropouts. In addition, the primary analysis and all sensitivity analyses also will be performed in the "per-protocol" sample.

#### Safety Analyses

Adverse events are classified using the MedDRA system [35] and categorized by system/diagnosis group, severity, relationship to study drug, action taken, and outcome. Tabulations will be prepared showing number and percent of subjects affected by an adverse event by treatment group, seriousness, relatedness, and level of cumulative exposure. The safety sample will be used for this analysis.

### Study Power

**Figure 1:**
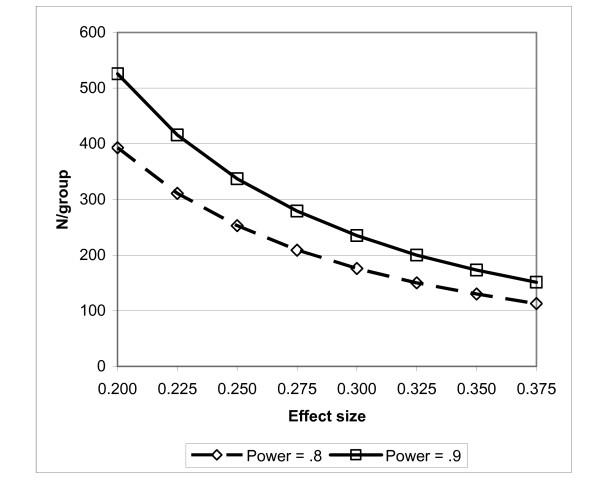
**Sample size needed at varying effect sizes, using normalized Poisson data**.

The power analysis takes account of the fact that we plan to transform the observed caries- increment scores to a normal distribution. We used a data simulation to determine what the impact of the transformation would be on the target effect size of 20% reduction in net caries increment. The results of our sample-size modeling, for varying effect sizes (Cohen's *d*, i.e., the ratio of mean difference to standard deviation (SD)), are shown in Figure 1. The 20% target effect size--expressed in rank-transformed units--is a *d *of 0.225, at which 416 randomized per group yields a power of 90%. The final target sample size of 1000 (500 per group) was deemed sufficient to provide adequate power, given what we believe to be conservative estimates of the effect size. These calculations do not adjust for attrition since we plan to use multiple imputation to obtain plausible replacement values for missing outcome measures.

## Discussion

Adult dental caries is an expensive and chronic condition for many Americans, particularly certain minority populations. PACS will provide useful data upon which health-care policy makers and the dental community can base decisions about whether to regularly include the use of chlorhexidine coating to decrease or prevent caries in adults. Our study will also analyze whether this treatment can be used cost-effectively in a variety of settings. The latter feature is particularly important given the present state of knowledge, as few dental preventive interventions have been appraised in terms of the economic implications of their cost and their benefit.

The PACS population is highly diverse, both in terms of the participants themselves and the dental delivery systems from which they were recruited. In the study's Navajo and Hopi population in the Southwest, no out-of-pocket direct payments are made by clients and indemnity is explicitly assumed by the care system; in the Pacific Northwest, the system is a not-for-profit dental Health Maintenance Organization (HMO) catering primarily to a white population that enjoys dental insurance coverage through employers; and in Massachusetts, the population is a mixed group served by a large dental insurance carrier under various service schema, as well as a study group recruited from Greater Boston by a large university dental clinic serving a primarily uninsured population. While these groups will be pooled for the primary analysis, secondary analyses will look at treatment effects in selected subgroups.

We identified several priorities in creating the PACS taxonomy for classifying dental caries. Priorities were creating the taxonomy needed to recognize the growing focus of dentistry on managing the non-cavitated lesion; being primarily visual and requiring only gentle probing to determine cavitation or the texture of the lesion base; being easy to use and reproducible; being backwardly compatible with conventional systems (e.g., Radike, World Health Organization, NIDCR); and avoiding distinguishing between active and non-active lesions to improve reliability of examiners. The resulting PACS taxonomy allows for identification of root carious lesions using a D1-D2 classification within the ICDAS descriptors, as well as allowing for identifying caries lesions associated with existing restorations for both coronal and root caries. This system is being used successfully in a separate, ongoing controlled clinical trial of the chlorhexidine coating in four American Indian communities.

Very few longitudinal studies of adult caries (1-4) have been conducted over the last 10 years. The use of D1 lesions in this trial will test the expanded visual diagnostic thresholds of ICDAS II in a large-scale randomized clinical trial. It is theorized that the use of D1 lesions to evaluate caries progression will permit shorter trials with fewer participants and better subject retention. If successful, such use will have implications for future dental research evaluating dental therapies. The PACS study is the first Phase III clinical trial to test this new model.

The need for new, cost-effective treatments to prevent or hinder the progression of adult caries is considerable. This study, together with other ongoing and completed trials of a chlorhexidine coating, is an important contribution to the establishment of sound evidence regarding the efficacy of this promising caries-prevention intervention.

## Competing interests

The study was initially conceived by CHX Technologies Inc., which recruited the study investigators and actively participated in the protocol development. Although primary funding for the study came from the National Institute of Dental and Craniofacial Research (NIDCR), CHX Technologies Inc. did provide the study treatments as well as financial support for some aspects of the protocol, including full support for the planned cost analysis (funding to JLF) and limited funding to support the microbiology substudy (funding to ASP). The study will also be used by CHX Technologies Inc. as a "pivotal" study under US Food and Drug Administration IND #45,466. Although CHX Technologies Inc. participated in Steering Committee discussions, it was not a voting member of the Steering Committee and is not part of any writing committees.

Apart from the above involvement by CHX Technologies Inc., the authors have no financial or non-financial competing interests to declare relative to this manuscript.

## Authors' contributions

All of the listed authors substantively contributed to the design or conduct of the study, participated in the preparation of this manuscript, and approved the final version of this manuscript. WMV, ASP, JDB, GM, CMG, JH, JSS, JLF, and BAW contributed to the study conception and design. ASP, GM, JH, JLF, RLL, and BAW contributed to the acquisition of data. WMV, CMG, and JH analyzed and interpreted data. CMG provided statistical analysis. WMV, ASP, CMG, and RLL drafted the manuscript. ASP, JDB, GM, CMG, JH, JSS, JLF, RLL, and BAW critically revised the manuscript. ASP, GM, JH, JSS, JLF, and RLL provided administrative, technical, or material support. WMV, JH, JLF, RLL, and BAW provided supervision. WMV, ASP, JH, JLF, and BAW obtained funding.

## Pre-publication history

The pre-publication history for this paper can be accessed here:

http://www.biomedcentral.com/1472-6831/10/23/prepub
